# Sleep deprivation and dendritic architecture: a systematic review and meta-analysis

**DOI:** 10.1093/sleep/zsaf146

**Published:** 2025-06-04

**Authors:** Alvin T S Brodin, Franziska Liesecke, Julia Spielbauer, Tobias E Karlsson

**Affiliations:** Department of Neuroscience, Karolinska Institutet, Solna, Sweden; Department of Plant Biology, Swedish University of Agricultural Sciences, Uppsala, Sweden; Department of Neuroscience, Karolinska Institutet, Solna, Sweden; Department of Neuroscience, Karolinska Institutet, Solna, Sweden

**Keywords:** sleep deprivation, synaptic plasticity, dendritic plasticity, gentle handling, dendritic architecture

## Abstract

Sleep is a well-conserved behavior, yet the functions of sleep remain uncertain and controversial. The synaptic homeostasis hypothesis proposes a central role for sleep, predicting that global synaptic strength increases after sleep deprivation (SD). Many studies have found changes in neuronal architecture following SD, but findings vary widely. This study provides the first systematic review of the effects of SD on dendritic architecture. We searched MEDLINE and Web of Science for rodent studies which reported dendritic spine density and/or dendritic length after SD compared to control. A total of 5090 records were screened, yielding 30 full texts for this meta-analysis. Studies were individually small and suffered from poor reporting regarding handling of data. Variability in structural measures was high between studies, indicating substantial methodological differences. We therefore developed a protocol for quality assessment of SD and spine/dendrite analysis, which can serve as framework for future studies. We also simulated experiments based on the included studies and showed that small sample sizes result in an overestimation of effect sizes. We conclude that current evidence does not support an effect from 24 hours or less of SD on dendritic structure. Chronic SD protocols of 72 hours or longer causes a decrease in Cornu Ammonis 1 (CA1), both in spine density and dendritic length, but it remains unclear whether this is a result of sleep loss or protocol-induced stress. This study provides a valuable overview of a field marked by conflicting findings, and clarifies which issues prevent robust conclusions from being drawn. Further progress in this field requires more robust handling of multi-level data, clearer guidelines on dendritic structure measurements and substantially higher-powered studies.

## Graphical Abstract

**Graphical Abstract:**
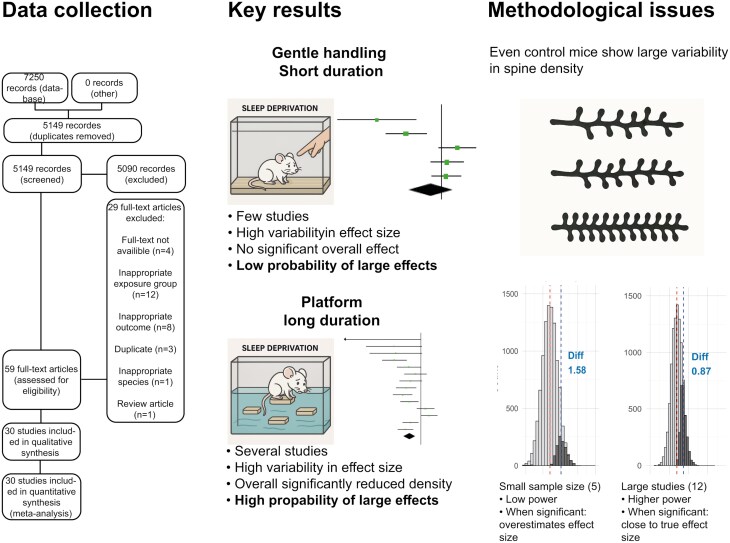

**Statement of Significance**

This systematic review and meta-analysis synthesizes current knowledge on changes in neuronal structure following short- and long-term sleep deprivation (SD). Our findings indicate that short-term SD has minimal impact on neuronal structure, whereas long-term SD leads to significant reduction in spine density. Notably, these effects are strongly associated with SD methods that are likely to impose substantial stress on the subjects. Furthermore, our analysis highlights that many studies to date lack sufficient statistical power to detect potentially meaningful changes. Additionally, the small sample sizes used in these studies increase the risk of overestimating the observed effects.

## Introduction

Sleep is a costly behavior that is highly conserved across the animal kingdom, yet its functional role remains only partially understood [1]. What is much clearer are the consequences that result from sleep deprivation (SD). Even a single night of insufficient sleep can significantly impair cognitive functions, leading to reduced attention, impaired memory function, and altered mood [1–3].

Poor sleep is not a new phenomenon. It has plagued humanity since biblical times [2]. Our modern lifestyles may have exacerbated this problem, since both shift work and intrusive technologies have the potential to disturb our natural rhythms [3], although the impact of this is disputed [4]. Impaired sleep quality and quantity has far-reaching effects on the body. It impacts the metabolic and cardiovascular systems with increased risks for cardiovascular disease and type 2 diabetes mellitus [5–7], as well as the immune system causing increased inflammatory cytokine production [8]. In the brain, SD decreases the clearance of soluble proteins, metabolites, and other waste products that were accumulated during the wakeful state, potentially contributing to neurodegenerative diseases such as Alzheimer’s disease [9, 10]. Sleep disturbances are also a common feature of other brain disorders, including anxiety, depression, and schizophrenia [11]. Thus, the importance of sleep is undisputed. But exactly what functions are performed by sleep is still far from clear and many controversies exist within the field. Without understanding what functions are performed during sleep and how they are governed, we are not likely to come closer to treating the damage caused by sleep loss.

An influential theory regarding the function of sleep is the synaptic homeostasis hypothesis (SHY). This theory posits that during wake there is a net increase in synaptic weights, which must then be compensated by downscaling during sleep [12]. Lack of sleep would then impair consolidation of prior experiences, as well as prevent proper encoding of future events due to a decreased signal-to-noise ratio [12].

Memory is indeed impaired by sleep loss. Sleep loss either before [13] or after [14] an event impairs recollection of that event. Sleep consists of both non-rapid eye movement sleep (NREM) and rapid eye movement sleep (REM), and both have been shown to be important for memory function. Sharp wave ripples, an electroencephalographic phenomenon occurring during the deeper stages of NREM sleep called slow wave sleep (SWS), correspond to hippocampal reactivation of memories and contribute to their consolidation [15, 16]. SWS is also important prior to learning; SWS deprivation has been found to impair subsequent memory formation even with preserved total sleep duration [17]. Once thought to be of lesser importance, REM sleep has been found to have important roles in memory formation. While some forms of learning are seemingly unaffected by selective REM-SD [18] contextual fear conditioning is clearly impaired [19], possibly reflecting a role for REM theta waves in consolidation of contextual memories [19]. These conclusions must however be caveated by the fact that “selective” REM-SD affects subsequent NREM sleep as well through the phenomenon of REM rebound, which increases the proportion of REM sleep to the detriment of NREM sleep [20]. Live-imaging studies have found that dendritic spines, which > 90% of excitatory synapses terminate on [21], are selectively maintained during both NREM [18] and REM sleep [22].

But the other key prediction of SHY, that synapse numbers and weight should increase without sleep, has been harder to verify. Some lines of indirect evidence exist. SD lowers seizure thresholds in both humans [23] and animal models [24], but other mechanisms such as increased inflammation could be at play here as well. In vivo two-photon imaging has demonstrated an overall decrease in spine size and GluA1 content during sleep, supporting SHY [25]. Finally, phosphorylation of plasticity-related proteins cycle during sleep–wake in a manner suggestive potentiation during wake and downscaling during sleep [26]. However histological studies after SD offer conflicting views. Hippocampal dendritic spine density has been found to either increase [27], decrease [28, 29], or remain unchanged [30] after SD. These discrepancies extend to the dendritic tree at large, with some studies reporting substantial decreases in dendritic length overall, which with a maintained or decreased spine density would greatly diminish overall synapse numbers [28]. Resolving these discrepancies is important for assessing the validity of SHY and for furthering our understanding of the health consequences of sleep loss; potential treatments clearly differ depending on if the problem is an excess or a deficit of synapses.

With such discrepant results stemming from rather similar interventions, it is crucial to move beyond individual studies and take a broader perspective by integrating all available data on how SD affects dendritic architecture. In this study we have systematically collected published studies that have assessed changes in dendrite length or spine density after SD and pooled them in a meta-analysis. Our aim is to assess whether a more conclusive answer regarding the structural effects of SD can be discerned, or if not, what heterogeneities explain the discrepancies in the literature. To achieve this, we conducted the first systematic review of the effects of SD on dendritic structure, resulting in a meta-analysis of 30 studies. This allows us to synthetize the currently available evidence on SD and dendritic structure. Several important aspects need to be improved to move research on structural plasticity forward in a more conclusive manner. We therefore developed a quality checklist to aid the design of robust studies on structural plasticity. Lastly, simulation experiments were performed to show the importance of having a proper sample size to have both power but also to detect the correct effect size.

## Methods

### Systematic review protocol

A systematic review protocol (CRD42022222584) was finalized and submitted to PROSPERO prior to the start of abstract screening and was accepted by PROSPERO during the abstract screening.

During the abstract screening, the decision was made to consider a more liberal pooling of studies including from different brain areas, which was precluded in the original protocol. This was to test for the a priori possible hypothesis that SD has universal effects across brain regions and treatment regimens. Further, a few studies were encountered in which animals were sleep deprived and then allowed some duration of recovery sleep before brains were harvested. The inclusion criteria were modified to exclude recovery periods deemed likely to mitigate the effects of the SD. Additionally, a quality control checklist was developed as the existing ones were not quite adapted to the specific methodology involved in this field. No other significant deviations from the strategy outlined in the protocol were made.

### Search strategy

The search strategy was developed in collaboration with Karolinska Institutet Library, based on a key set of studies that met the inclusion criteria. The initial search was performed on February 17, 2021, and updated August 8, 2024.

The following search strings can be used to replicate the searches:

Pubmed: (“Sleep”[MeSH] OR sleep*[tiab] OR (dyssomnia*[tiab] OR “dys-somnia”[tiab] OR insomnia*[tiab])) AND (“Dendrites”[MeSH] OR dendrit*[tiab] OR synaptic*[tiab] OR “sholl analys*”[tiab])

Web of Science: TS = (sleep* OR dyssomnia OR dys-somnia OR insomnia) AND TS = (dendrite* OR dendritic* OR synaptic* OR (sholl NEAR/2 analys*))

### Record screening and selection

Results of the literature search were imported to covidence.

Screening of papers was performed in two stages as detailed below.

Title/Abstract screening was performed independently in duplicate by two of the authors of this paper using Covidence. If the assessments differed at this stage between the researchers, the paper went on to full-text screening.

For studies that did not meet any exclusion criteria for the title/abstract screening as specified above, or where a definitive decision could not be made from the title/abstract, full texts were retrieved and screened. All studies that did not meet any exclusion criteria in the full-text screening were included in the review.

### Inclusion and exclusion criteria

We included peer-reviewed controlled original studies investigating the effects of SD on dendritic length, spine density, and/or spine formation and elimination. We restricted the study population to mice or rats, of any age, gender, or strain. There had to be an intervention group that was sleep deprived, partially or fully, for at least 3 hours, and a corresponding control group that was allowed to sleep.

To this effect, the following exclusion criteria were applied at each stage of record screening:

#### Title/Abstract screening

1. Article type: Paper is a review or opinion piece.
2. Species: Not a study on mice or rats.
3. Sleep: Does not study effects of sleep or SD.
4. Structure: Does not study dendritic structure, spine numbers, or synapse numbers.

#### Full-text screening

5. Availability: Full text could not be obtained.
6. Language: Not available in English, German, French, or Swedish.
7. Exposure group: Does not contain a group which was sleep deprived, fully or partially, with any recovery sleep not exceeding 1/7 of the SD period.
8. Control group: Does not control a group which was not sleep deprived.
9. Outcome: Does not report at least one of the below for both the exposure and control group:

a. Spine density,
b. Dendritic length, and
c. Rate of spine elimination and formation.

10. Duration: Animals were sleep-deprived for less than 3 hours.

### Data extraction

The following data items were extracted from all included articles: “Title,” “First author,” “Publication year,” “Power analysis,” “Species,” “Strain,” “Animal Supplier,” “Genetic Modification,” “Sex,” “Age,” “Cage group size,” “Light Cycle,” “Feeding,” “Handling,” “SD method,” “SD duration,” “SD validation,” “Other animal interventions,” “Tissue fixation and staining,” “Imaging method,” “Tracing method,” “Tissue shrinkage.”

For each outcome measure, the following data items were collected for each treatment and control group (where applicable): “Treatment,” “Outcome measure,” “Group size” “Brain region,” “Cell type,” “Dendrite type,” “Spine type,” “Mean,” “standard deviation,” “confidence interval,” “Statistical method,” “*p*-value,” “Number of neurons imaged,” “Number of dendrite segments imaged,” “Order of dendritic branches analyzed.” Spine density was recalculated to “spines per 10 µm” and dendritic length to “µm.”

If any of the above data could not be determined from the full text, first the supplemental data of the paper was consulted, and if there was still uncertainty the corresponding author of the paper was contacted via email to request clarification. When data was not presented numerically, mean and variance were estimated from figures using WebPlotDigitizer.

Where articles only reported standard error (SE), the standard deviation was recalculated using the reported n for each group. In some cases, both the number of animals and neurons were listed, and if unclear which had been used to calculate SE the number of neurons was used.

Where articles only reported median and range, mean and standard deviation were estimated by assuming the mean is equal to the median, and that the standard deviation is equal to the range/4, or range/6 if there are any outliers. This method will be inaccurate especially if the data is not normally distributed, but was deemed to still introduce less bias than excluding these studies.

### Data synthesis

Quantitative synthesis was considered for all outcome measures reported by at least three studies. Subgroup analysis was performed according to the following stratifying factors where sufficient (3+) studies were available.

- Acute (3–24 hours) or chronic (> 72 hours) SD,
- Species,
- Brain region,
- SD method, and
- Age of animals.

Effect measures were converted to standardized mean differences (SMD), calculated as Cohen’s *d*. Heterogeneity was assessed using the *I*^2^ statistic (with 0%–30% interpreted as indicating low heterogeneity, 30%–60% moderate heterogeneity, and 60%–100% substantial heterogeneity).

For each outcome, a random effects model was used for the meta-analysis as considerable heterogeneity is expected between studies.

Analysis was performed in Cochranes Review Manager 5.4.1. Meta-analysis is presented as forest plots of SMD, with included studies arranged according to weight, with highest weight at the bottom of the forest plot.

Jason Griffin’s Metapower application (https://jason-griffin.shinyapps.io/shiny_metapower/) was employed for power calculations.

Several studies include multiple independent datasets eligible for inclusion. Each dataset was included when applicable, but never more than once per subgroup to prevent any single study from being overrepresented. When multiple datasets from the same study were eligible for the same subgroup, the dataset with parameters most aligned with the majority of other studies in that subgroup was selected.

### Quality assessment

The quality of the original articles included in this meta-analysis was evaluated using two different protocols. First, papers were screened using the ARRIVE Essential 10 guideline published in 2020 in PLOS Biology [31]. The checklist can be used to analyze transparency and quality of reporting in a study. Secondly, we developed a risk of bias tool inspired by SYRCLE [32] specifically for studies investigating the effect of SD on dendritic structure. This risk assessment protocol focuses on potential sources of bias in animal allocation, stress induced by the SD protocol, transparent selection of neurons for analysis, and robust use of statistics on dendritic measurements. Based on the risk of bias tool, hierarchical cluster analysis was performed on the studies using the complete linkage method. Risk assessment was performed by two researchers independently, with disputes resolved by a third researcher.

### Simulation

To assess how the design of future studies could be optimized we simulated data based on the median standard deviation from studies using the Golgi method in CA1 (the largest group). We simulated the power achieved when using different numbers of animals *n* = 5 (median from this data) and 12 (max in the dataset), and effect sizes 10%, 20%, and 30% using 10 000 simulations per datapoint.

## Results

### Included studies

**Figure 1.**
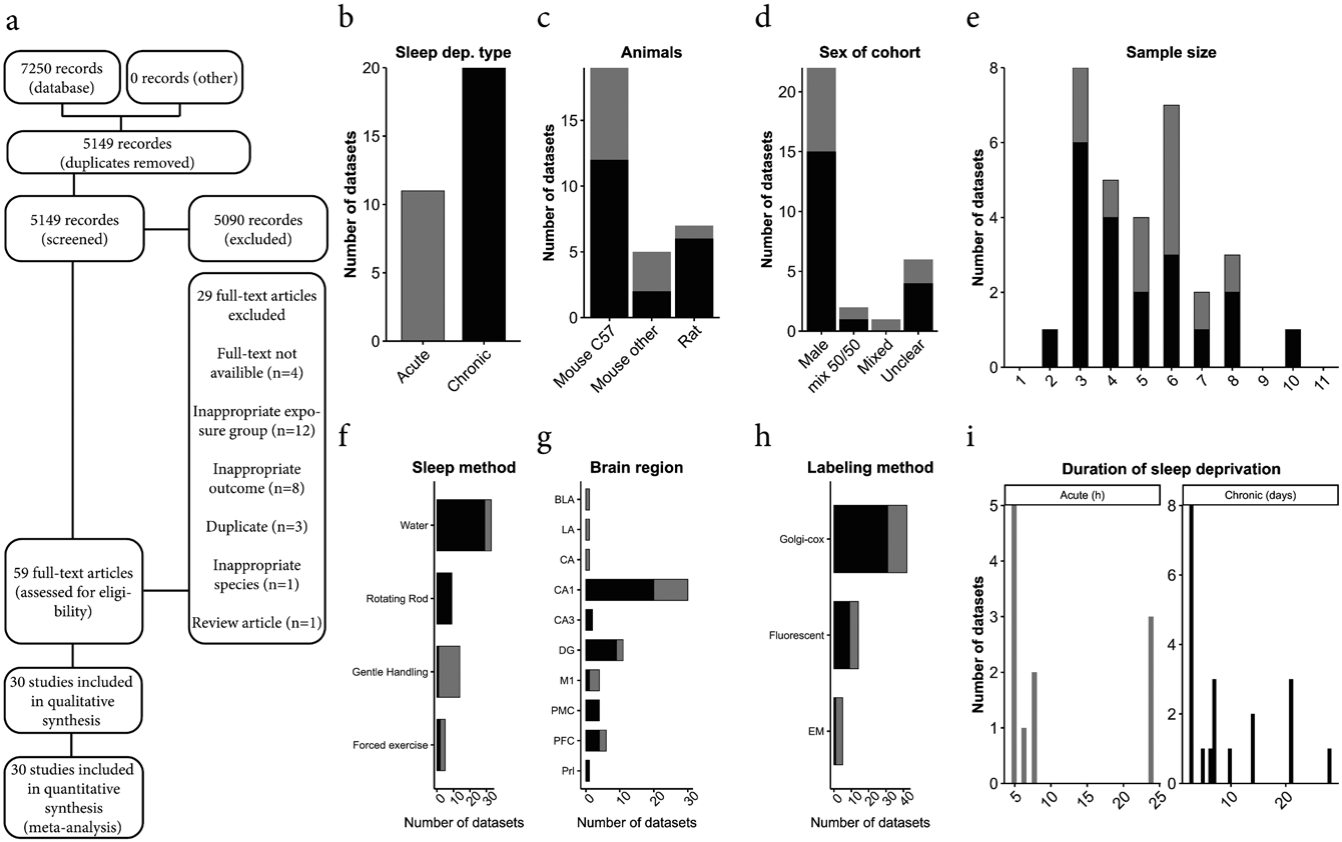
Study selection diagram and basic characteristics. a. Flow chart of the study selection process. b. Distribution of acute (≤24 hours, gray) or chronic (> 72 hours, black) SD protocols. c. Distribution of animal species and strain. d. Sex distribution of experimental animals. e. Distribution of sample sizes per experimental group. f. Distribution of SD methods. g. Distribution of examined brain regions. h. Distribution of neural labeling methods. i. Distribution of SD durations.

In total, 7250 records were identified through database searches, which rendered 5149 records after removal of duplicates. Of these, 5090 records were excluded through abstract screening, and a further 20 articles after full-text screening, leaving 30 articles to be included in this review, all in English (Figure 1a). Study characteristics are summarized in Table 1 and illustrated in Figure 1.

**Table 1.** Characteristics of included studies

Study	SD protocol	SD duration	Brain region(s)
Acosta-Pena et al. [33]	Gentle handling	24 h	CA1 and PFC
Brodin et al. [30]	Gentle handling	5 h	CA1
Chen et al. [34]	Water-filled cage	5 days	CA1 and PMC
Cheng et al. [35]	Treadmill	18 h per day for 3 days	PFC
Gao et al. [36]	Platforms in water	20 h per day for 10 days	CA1
Giri et al. [37]	Flower-pot	6 days	CA1 and CA3
Gisabella et al. [27]	Gentle handling	5 h	CA1
Havekes et al. [28]	Gentle handling	5 h	CA1
Gao et al. [38]	Rotating Rod	21 h per day for 7 days	DG
Jia et al. [39]	Platforms in water	20 h per day for 2 weeks	DG
Jiao et al. [40]	Platforms in water	24, 48, or 72 h	CA1
Kim et al. [41]	Platforms in water	3 days	CA1
Kim et al. [42]	Platforms in water	3 days out of 7 for 4 weeks	CA1
Li et al. [43]	Platforms in water	96 h	DG
Li et al. [44]	Rotating rod	20 h per day for 1 week	CA1
Muzio et al. [45]	Rotating rod	3 days out of 5	DG
Nagai et al. [46]	Running wheel	6–7 h or 5 days	M1
Noorafshan et al. [47]	Platforms in water	18 h/day for 21 days	CA1
Raven et al. [29]	Gentle handling	5 h	DG
Rexrode et al. [48]	Rotating rod	5 h	Amygdala
Spano et al. [49]	Gentle handling	6–8 h	CA1
Tabassum et al. [50]	Treadmill	72 h	PFC
Tuan and Lee [51]	Platforms in water	72 h	DG
Tuan et al. [52]	Platforms in water	72 h	DG
Wang et al. [53]	Platforms in water	72 h	CA1
Xin et al. [54]	Running platform	8 h	DG
Zhang et al. [55]	Platforms in water	72 h	CA1
Zhang et al. [56]	Platforms in water	24 h	CA1
Zhang et al. [57]	Platforms in water	20 h per day for 3 weeks	CA1
Zhu et al. [58]	Platforms in water	20 h per day for 2 weeks	PrL

### Characteristics of studies

We found that SD was split into studies looking at the effects of a single instance of sleep loss and studies that looked at longer periods of SD.

SD protocols were therefore split into “acute SD” of 24 hours or less (*n* = 10, 33%) or “chronic SD” of 72 hours or longer (*n* = 18, 60%) (Figure 1b), with two studies studying both acute and chronic SD. Out of the 12 acute SD protocols, 9 involved durations between 5 and 8 hours, while the chronic SD protocols ranged from 72 hours to 4 weeks (Figure 1i). Most studies were performed in mice (*n* = 23, 77%) with mice from a C57bl6 background being the most common (sub-strains where pooled for Figure 1c), with relatively few rat studies (*n* = 7, 23%). Almost all studies (*n* = 23, 77%) restricted the study population to males, with two studies having a mixed sex population and five not clearly reporting sex distribution of the included sample (Figure 1d). Sample sizes ranged from 4 to 12 animals per group, with an average group size of 5 (Figure 1e).

The most common method of SD was variations of the multiple platforms in water approach (*n* = 15, 50%), followed by gentle handling (*n* = 7, 23%) and rotating rod (*n* = 3, 10%) (Fig. 1f). Only two studies used spinning disk or rotating cage setups (*n* = 2, 7%), while a single study housed mice in a water-filled cage (*n* = 1, 3%). Chronic SD was most commonly achieved by variations on the platforms in water approach (*n* = 14, 70%), with two studies using some form of forced exercise (treadmill or spinning disk), two a rotating rod, a single study using a water-filled cage and a single study employing a mix of novel object and gentle handling. Acute SD was widely achieved by gentle handling (*n* = 8, 67%), with two studies using platforms in water, one study using a rotating rod and one study employing a mix of novel object and gentle handling.

The hippocampus was studied in 26 of 30 studies (87%), divided between CA1 (*n* = 18, 60%), dentate gyrus (*n* = 8, 27%) and CA3 (*n* = 1, 3%) (Figure 1g). Other investigated brain regions were motor cortex (*n* = 2, 6%), prefrontal cortex (*n* = 2, 6%), amygdala (*n* = 1, 3%), and prelimbic cortex (*n* = 1, 3%) (Figure 1g). Note that some studies investigated more than one area. Golgi staining was the most prevalent visualization method (*n* = 22, 73%), followed by fluorescent protein either using transgenic mice or viral vectors (*n* = 5, 17%), electron microscopy (*n* = 2, 7%), and intracellular dye injection (*n* = 1, 3%) (Figure 1h).

Most studies measured spine density (*n* = 21, 70%), with eight (27%) also reporting dendritic length. A single study only measured dendritic length. No studies which measured formation/elimination of spines met the inclusion criteria.

In summary, we can say that two bigger groups of studies emerged; the most common SD method was chronic SD using the multiple platforms in water approach. The second group was acute SD predominantly utilizing gentle handling. Studies where almost exclusively performed in male animals with a majority using six or fewer mice per group. Regarding the assessment of changes in synaptic structure, the CA1 and dentate gyrus (DG) areas of the hippocampal formation were the by far most assessed with a few studies looking at the cortex and a single study investigating the amygdala. The most common method used to visualize the structure of neurons was Golgi-Cox, with a few studies using fluorescent proteins. Only a single study used electron microscopy (EM ).

### SD and dendritic length

We started by reviewing studies that examined alterations in dendritic structure resulting from SD. Changes in dendritic structure following SD could have dramatic effects on the function of the neuron by changing both lengths where synapse can be formed but also changing location of the dendritic tree in relation to incoming axons thereby changing what input will drive the firing of that neuron. As described later, the precise location of synaptic changes was not consistently reported hence no analysis of the potential input affected could be performed. Ten studies were included that recorded dendritic length, three after acute SD and seven after chronic SD. Some of these recorded dendritic length in µm, while some noted it as a percentage of dendritic length in free-moving controls. All of these were deemed suitable for inclusion in a meta-analysis by two independent researchers.

**Figure 2.**
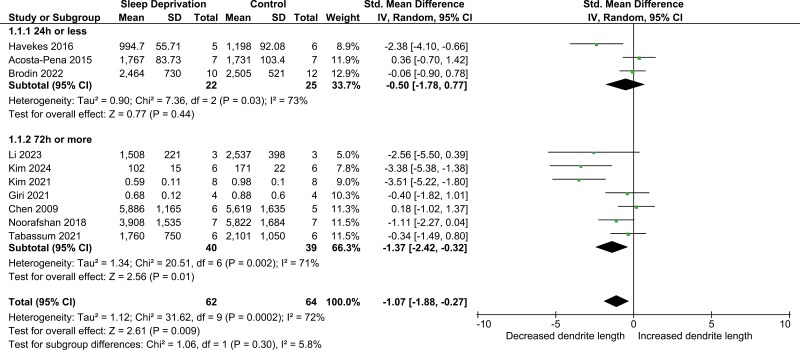
Forest plot of dendritic length by SD duration. The studies are ordered within each subgroup with the highest weighted studies towards the bottom. The size of the dot (green) reflects study weight, error bars indicate 95% CI, and black diamonds indicate 95% CI of summary effect.

We began by looking at an overall change in dendritic length using all the studies included and found a significant reduction in dendritic length (SMD −1.07; 95% confidence interval [CI] −1.88, −0.27). While this group includes quite disparate protocols, we did not want to a priori discount the possibility that SD had a similar effect across these protocols. Next two main types of SD, acute (< 24 hours) or chronic (> 72 hours), were investigated. This subgroup analysis revealed a difference in effects on dendritic length between acute and chronic SD. The chronic group had a significant reduction of dendritic length (SMD −1.37; 95% CI −2.42, −0.32). This corresponds to a decrease by 20%–40% of the measured dendritic tree. In contrast, acute SD had no significant effect on dendritic length (SMD −0.50; 95% CI −1.78, 0.77) (Figure 2). However, there was no significant difference between the subgroups (*p* = .30) (Figure 2). These outcomes were invariant upon exclusion of any single study.

We next assessed the power achieved by our meta-analysis. Effect sizes in the included studies were often large, with multiple studies reporting decreases or increases in dendritic length by more than 30%, corresponding to an SMD greater than one. For SMD > 1 the meta-analysis had > 79% power when pooling each of the studies (Supplementary Figure S1A). However, the low number of animals per study, and the high heterogeneity, means that power to detect smaller differences is severely lacking, with 29 studies required to reach 80% power to detect a moderate effect size of 0.5 SMD (Supplementary Figure S1B). Consequently, the power of the subgroup analyses is also severely lacking.

Heterogeneity was substantial between studies both overall (*I*^2^ = 72%), for the acute subgroup (*I*^2^ 73%), and for the chronic subgroup (*I*^2^ 71%) (Figure 2), suggesting that a large part of the variability between studies could result from changes between methodologies and populations of the studies. However, due to the low number of studies investigating dendritic length we did not have sufficient power to perform further subgroup analysis.

We further looked at the distribution of CA1 dendritic lengths in control animals across the studies where absolute measurements were provided (Supplementary Figure S2). There was a considerable range of estimates, from > 5000 µm to ~1000 µm, although most studies were in the range 2000–2500 µm. The small number of studies limits what conclusions can be drawn, but no obvious effects from either staining method or species can be detected.

We can conclude that there is a significant decrease in dendritic length between SD and control group overall and after chronic SD thanks to large effect sizes. No significant effect was found after acute SD, but the low number of studies mean we cannot exclude biologically significant changes in dendritic length, as even large decreases or moderate increases fit the limited data included. Similarly, we cannot conclude either that there is a true difference between acute and chronic SD in its effect on dendritic length.

### SD and spine density

Spine density was assessed in most of the included studies resulting in that 29/30 studies were included in this meta-analysis. Most common was to report spine density per 10 µm however other units were also used (1 µm or 100 µm).

**Figure 3.**
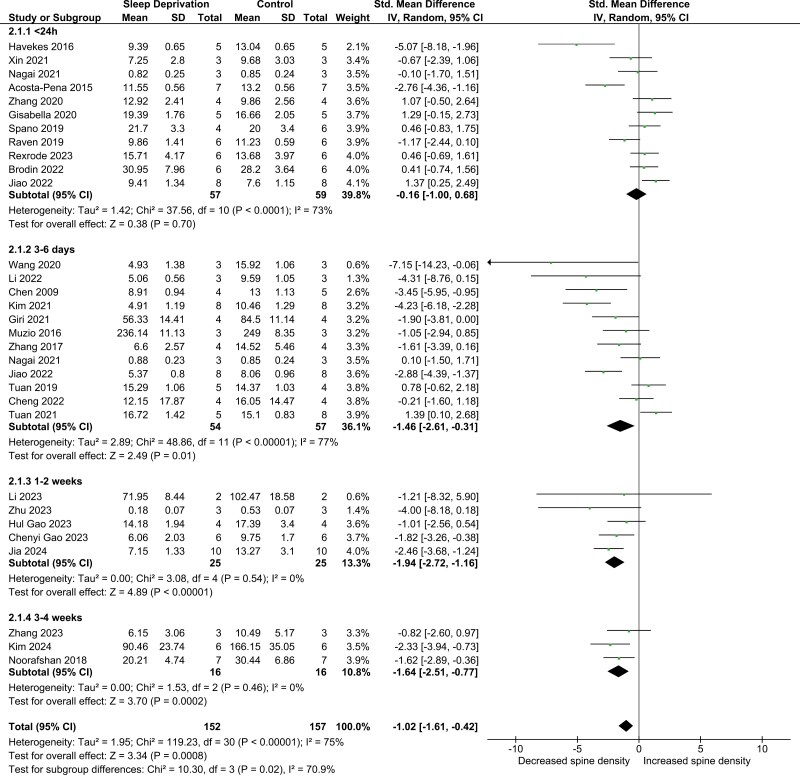
Forest plot of spine density by SD duration. The studies are ordered within each subgroup with the highest weighted studies towards the bottom. The size of the dot (green) reflects study weight, error bars indicate 95% CI, and black diamonds indicate 95% CI of summary effect.

We hypothesized that effects on spine density should increase with a prolonged duration of SD. With a larger number of studies and a wider range of durations it was deemed appropriate to stratify within the chronic SD studies. Therefore, we analyzed the effect on spine density in subgroups in accordance with SD duration (Figure 3). There was a significant decrease in dendritic spine density overall (SMD −1.02; 95% CI −1.61, −0.42) as well as for the subgroups 3–7 days of SD (SMD −1.46; 95% CI −2.61, −0.31), 1–2 weeks (SMD −1.94; 95% CI −2.72, −1.16) and 2–4 weeks (SMD −1.64; 95% CI −2.51, −0.77) (Figure 3). Twenty-four hours or less of SD did not result in any significant change in spine density (SMD −0.16; 95% CI −1.00, 0.68) (Figure 3). These outcomes were invariant upon exclusion of any single study. Heterogeneity was substantial overall (*I*^2^ = 75%) and within the < 24 hours (*I*^2^ = 73%) and 3–7 days (*I*^2^ = 77%) subgroups, while it was negligible within the 1–2 weeks (*I*^2^ = 0%) and 2–4 weeks (*I*^2^ = 0%) subgroups (Figure 3).

Quite disparate methods of SD were used across these studies, some involving aversive stimuli, some involving forced exercise. Hence, we wanted to see if the methods used resulted in different effects on spine density. We therefore stratified the studies according to the SD method (Supplementary Figure S3). The various variations on the multiple platforms in water method were merged with the studies using shallow water in the cage, as they shared a common methodology in REM-sleep atonia causing submersion in water. The water subgroup was by far the biggest and consisted of only studies that used chronic SD. In this group there was a significant reduction in the density of dendritic spines with a very large effect size (SMD −1.70; 95% CI −2.62, −0.78) (Supplementary Figure S3). The gentle handling method is much more labor intense than the other methods, hence it is no surprise that this method was not used for chronic studies, although a single study used it for several days. For acute SD the gentle handling method was the most common method. No significant reduction in dendritic spine density was found in the gentle handling group (SMD −0.69; 95% CI −1.90, 0.52) (Supplementary Figure S3). The final two methods, rotating rod and forced exercise, were mostly used for chronic SD. Neither rotating rod (SMD −0.28; 95% CI −1.14, 0.57) nor forced exercise (SMD −0.28; 95% CI −1.13, 0.66) had a significant effect on spine density (Supplementary Figure S3). Heterogeneity was substantial in both the gentle handling (*I*^2^ = 78%) and water (*I*^2^ = 75%) subgroups, while it was negligible in the rotating rod (*I*^2^ = 4%) and forced exercise subgroups (*I*^2^ = 0%), but the latter groups were too small to have any certainty in this measurement. Note that the difference between the gentle handling and water subgroups was not statistically significant.

The response to SD could vary in different regions of the brain with some areas being more susceptible than others. We therefore stratified the studies depending on the region analyzed (Supplementary Figure S4). CA3, amygdala, and prelimbic cortex were each investigated by a single study; their data is included in the figure for completeness, but these groups are too small to meaningfully interpret on their own. CA1 was the most analyzed region and here we found a significant reduction in dendritic spine density (SMD −1.40; 95% CI −2.31, −0.50) (Supplementary Figure S4). The second most common region to analyze was DG and here we did not find a significant effect (SMD −0.76; 95% CI −1.83, 0.32) (Supplementary Figure S4). We finally looked at all studies that had measured spine density in the frontal or prefrontal cortex and found no significant effect of SD. (SMD −0.66; 95% CI −1.36, 0.04), however this was dependent on the inclusion of Nagai 2021 (Supplementary Figure S4). Exclude that study and the effect reached statistical significance (SMD −0.83; 95% CI −1.60, −0.06). Heterogeneity was substantial in the CA1 (*I*^2^ = 79%) and DG (*I*^2^ = 73%) subgroups, low in the frontal cortex subgroup (*I*^2^ = 25%) (Supplementary Figure S4).

**Figure 4.**
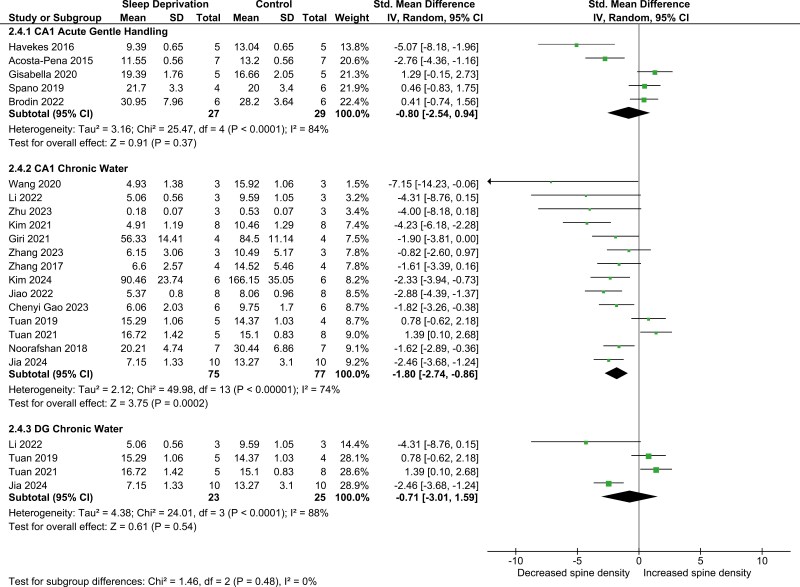
Forest plot of spine density, matched brain region, duration, and method. The studies were ordered within each subgroup with the highest weighted studies towards the bottom. The size of the dot (green) reflects study weight, error bars indicate 95% CI and black diamonds indicate 95% CI of summary effect.

Ultimately, given that the outcomes varied depending on brain region, SD method, and duration, we grouped these variables into three potentially more homogeneous subgroups (Figure 4). The first consisted of studies using gentle handling for < 24 hours and investigating spine density in the CA1 and here we found no significant effect on spine density (SMD −0.80; 95% CI −2.54, 0.94) (Figure 4). The second consisted of studies using a water-based method for 72 hours or longer, also assessing spine in CA1. In this subgroup, we found a significant and very large reduction in spine density (SMD −1.80; 95% CI −2.74, −0.86) (Figure 4). Finally, another subgroup used water-based methods for 72 hours or longer and investigated the DG. Here there was no significant effect on spine density (SMD −0.71; 95% CI −3.01, 1.59) (Figure 4). Heterogeneity remained substantial in all groups (*I*^2^ = 84%, 74%, and 88%, respectively) (Figure 4). There were no statistically significant differences between the subgroups.

Effect sizes were often large, with multiple studies reporting decreases or increases in spine density of 1–4 SMD. The power to detect an SMD of 1 in the pooled analysis was 98% (Supplementary Figure S1C), with 57% to detect an SMD of 0.5 (Supplementary Figure S1D).

**Figure 5.**
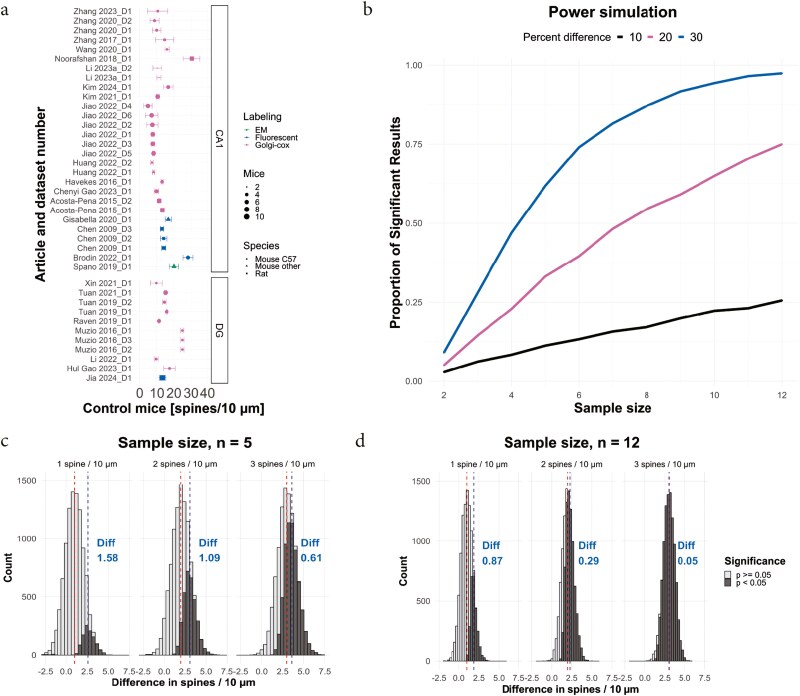
Variability in spine density between studies and power simulation. a. Average spine density and 95% CI for the control groups across included studies. Size of the dot reflects sample size, color the labeling method and shape of the dot the species. b. Power simulation using three effect sizes (10, 20, or 30% change in spine density, black, red and blue line, respectively). The dashed line shows the cumulative distribution of experimental animals across studies included in this review. c–d. shows the simulated outcome when performing 10 000 experiments and a *n* of 5 (c) and 12 (d).

As there was a substantial amount of heterogeneity indicating substantial differences between the studies, we next investigated the spine density in control mice. We hypothesized that different methods would result in significant differences in spine density where some methods such as EM or fluorescence imaging using confocal imaging would be able to find more spines as they allow detection above and below the dendrite. In several of the studies, the unit given was deemed to be highly unlikely and, in these cases, we changed to the unit that would give a spine density within the same order of magnitude as the rest of the studies. However, even after this correction, when we plotted the spine density in the different studies, we were surprised to see the large differences in spine density between different studies (Figure 5a). Looking only at mice from the C57bl6 strain (all sub-strains included), spine densities in CA1 ranged from 4.8 to 16.6 spines per 10 µm, with a mean of 10.2. Hence, when assessing control mice of the same strain the spine density varied by more than threefold. In DG the density ranged from 9.7 to 24.9 spines per 10 µm. Overall estimates of control spine density were with few exceptions only similar within control groups from the same study. No clear trend based on labeling method or brain region was found, nor was there a detectable effect of age on spine density (*R*^2^ = 0.001, *p* = 1, Supplementary Figure S6).

We next estimated the median standard deviation as a percentage of spine density in the CA1 when Golgi staining was used and found it to be 17.8%. Using this estimate, we simulated the power to detect changes in spine density with a 10% (small), 20% (medium), or 30% (large) effect size (Figure 5b). We used a range of mice per group from 2 (the lowest reported) to 12 using 10 000 samples per group and effect size. The power when using 12 mice per group was 97.6% to detect a 30% change, 75.1% for 20%, and 25.6% for a 10% change. In total, 78% of the studies included in this meta-analysis used 6 or fewer mice. The power when using 5 mice per group (the median number in the included studies) was 62.6% for a 30% change, 31.7% for a 20% change, and 11.7% for a 10% change. As the sample size in a group not only affects the change of a significant result but also the size of the effect you are likely to find, we performed simulations of experiments to estimate what effects that would be found to be significant depending on sample size. The spine density was set to 10 spines per 10 µm for the simulation. If five animals per group was used (the median of included studies) and the median standard deviation was used the average spine difference between control and experimental group was found to be 25.8% in the experiments that were significant compared to the 10% difference that was simulated, 30.9% when 20% was simulated and 36.1% when 30% was simulated (Figure 5c). Note that the total sample simulated had a mean very close to the selected difference in all cases. When the number of mice was increased to 12 the significant subpopulation increased as expected from the power simulation perform previously. Here the estimates were closer to the selected means.18.7% when a 10% difference was simulated, 22.9% when 20% was simulated and 30.5% when 30% was simulated (Figure 5d).

### Quality assessment

It is central to scientific progress that results from previous studies can be used when planning future studies. As a first step, it is vital that it is clear from the methods and reporting in a study what has been down and how. The ARRIVE Essential 10 guideline was published in 2020 in PLOS Biology and has been adapted by many journals to improve the reporting of animal experiments. We therefore began by assessing all studies using the ARRIVE guidelines [31]. The ARRIVE screen is visualized as a heatmap displaying included articles depending on clear reporting in relation to the checklist (Supplementary Figure S5). Studies very seldom reported if they had inclusion and exclusion criteria or even if inclusion/exclusion of study animals had occurred. Most studies were sufficiently transparent on basic characteristics of the experimental animals and reported clear study designs, as well as adequation description of statistical methods used. However, even though most studies describe the number of animals used in their study, only 3 out of 30 studies mention conducting any form of power calculation. 26.67% of the articles checked the normality or other requirements of their data before using statistical methods to compare control and sleep deprived groups. Only one study provided both effect size and CI. While no study fulfilled all the required parts of the ARRIVE essential, all included studies fulfilled more than half of the ARRIVE requirements.

**Figure 6.**
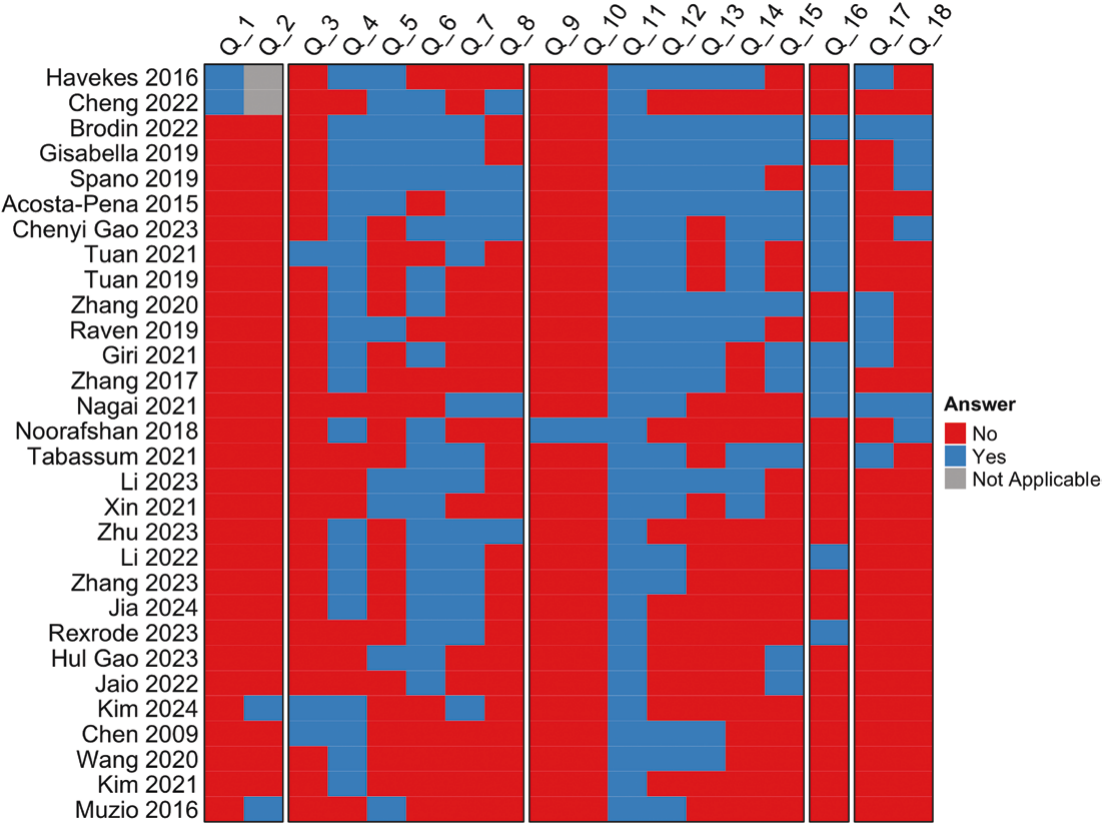
Methodological checklist outcomes. Green boxes indicate positive and red boxes indicate a negative answer to the question, with gray boxes indicating the question is not applicable. Questions 1–2 relate to matching baseline characteristics of animals, 3–8 SD protocol, 9–15 dendritic staining and imaging, 16 statistics, and 17–18 selection of neurons and dendrites for analysis. Detailed questions (Q1–Q18) of the checklist can be found in Table 2.

Secondly, we developed a methodological checklist for analyzing structural plasticity after SD (questions in Table 2 and detailed graph in Supplementary Figure S7, outcomes per question group in Figure 6). The protocol is intended to be useful for planning structural studies with clear guidelines for tissue processing, imaging, and analysis that are relevant to all studies, as well as a method-specific part that in this case is focused on SD. All categories of the protocol show mixed results. Papers were clustered based on which criteria they fulfilled. We analyzed them based on cluster, but the clustering was highly correlated to method and duration hence, it is not possible to draw a conclusion about the importance of the criteria in relation to the effects of duration and method (Supplementary Figures S8–S10). Only 4 (13%) studies reported adequate matching of baseline characteristics of control and SD animals, but in 19 (63%) studies SD and control mice were exposed to the same environment. Only 6 (20%) of studies monitored SD effectiveness. Only a single study (3%) reported measuring and compensating for shrinkage or expansion of samples. Only 3 (10%) studies measured stress levels of the mice. In almost all studies the specific brain region and cell type was clearly reported and when it was applicable. 40% described the type of dendrites and 43% stated the location of the dendrites used for measurements. Criteria to determine an eligible neuron and dendrite were mostly hidden from the reader. Furthermore, the minimal length of a dendrite to be counted and how that length was determined was also very rarely stated.

**Table 2.** Methodological checklist

Domain	Number	Question	Answer (Yes (1), No (0), Unclear (U), Not Applicable (N))
Baseline characteristics	1	Were littermates used as control?	
	2	If littermates were not used, did the investigators adequately adjust for unequal distribution of baseline characteristics between control and SD mice in the analysis?	
SD protocol	3	Was the level of stress measured that was caused by the SD protocol?	
	4	Could the mouse remain stationary during the whole SD protocol?	
	5	Were only non-aversive stimuli used in the SD protocol?	
	6	Were control and SD group exposed to the same environment?	
	7	Was resting period between SD and tissue collection specified?	
	8	Was SD effectiveness monitored?	
Staining/Imaging	9	Were changes in tissue size measured?	
	10	Were measurements normalized to compensate for changes in tissue size?	
	11	Is the exact brain region stated in which measurements were performed?	
	12	Is the cell type measured specified?	
	13	Is it stated if apical or basal dendrites were measured?	
	14	Is it stated to which branch order dendrites that are included belong to or is the location of the dendrites stated?	
	15	Is it stated how many neurons per mouse were measured?	
Statistics	16	Are different neurons from the same animal treated as dependent data points?	
Neuron inclusion/exclusion	17	Are the inclusion and exclusion criteria stated for neuron selection?	
	18	Is it stated how suitable dendrites were defined?	

In total, 19 (63%) studies did not clearly state if they treated neurons from the same animal as dependent data points. Out of these, seven left it ambiguous how they handled the data. Incorrectly treating neurons as independent data points would falsely inflate the apparent power of the study, increase the risk of false positive significant results and affect measures such as standard error of the mean Specifically for this meta-analysis, it would also lead to an underestimation of standard deviation, and consequently an overestimation of SMD. An underestimation of SD would also affect the power simulation. In the preceding analyses, we have attempted to correct this in the studies where it was apparent, but in the seven studies where statistical analysis was ambiguous, we assumed that the authors had handled the data appropriately. To investigate the consequences of this assumption, we reran the meta-analysis assuming that the data from those studies as well had been falsely inflated with an assumed five neurons per animal treated as independent data points. This lowered the effect size of the included studies as expected, but did not significantly affect the overall outcome of the meta-analysis (Supplementary Figure S8).

## Discussion

In this systematic review, we assess the evidence regarding structural changes in the dendritic tree caused by SD in rodents. To the best of our knowledge, this is the first systematic review on this topic. Determining what changes, if any, affect the dendritic tree after SD has important implications for our understanding of the function of sleep, the validity of SHY, and our assessment of the health risks posed by SD.

Several methodological issues were widespread and limited the conclusions that can be drawn. Our meta-analysis shows a shortage of reporting and transparency in most included articles when it comes to basic statistical requirements such as assessing the normality of data, power calculations, reporting of the effect size, and CI. Most of the studies were consistent with reporting on methods used for post-hoc comparisons. The ARRIVE Essential 10 guideline evaluates the essential minimum information needed for readers to assess the reliability of research findings and serves as a good guideline for reporting for future studies.

While a considerable number of studies were included in this meta-analysis, these were all individually small, limiting overall power. Owing to large effect sizes, post-hoc power was decent, but the ability to rule out smaller or even moderate changes especially after acute SD or in the less studied brain areas remains poor. Additionally, precisely because the included studies were small, it is likely the effect estimates are inflated. Small studies are known to be prone to significant overestimations of effect size via the “winner’s curse” phenomenon [59]. Our simulations show that this can have drastic effects especially when the effect and sample size is small. Hence, when studies use a low number of animals per group it is very likely that they will also overestimate the effect if it is significant. Very few studies performed any kind of power analysis. We have in this paper performed a power simulation with an easy-to-use R script. Future studies should either perform a power simulation or calculation of their own, or use the simulation performed in this paper for guidance.

Further, there is considerable uncertainty in the estimations of standard deviation of the included studies. Morphological studies of the dendritic tree typically involve tracing multiple neurons per animal. However, it is well known that these measurements are often erroneously treated as being independent of each other, leading to a downward shift of both *p*-values and standard deviation [60]. Most studies included in this review did not specify whether each neuron was treated as an independent datapoint, which created uncertainty in how to calculate the standard deviation from the provided SEs. Many studies reported the n as being equal to the number of mice used, and in the absence of other information this is the number assumed to have been used for SE calculation. We suspect, but currently have no way of knowing, that this reporting is misleading. The impact on this meta-analysis proved to be limited, but if these practices continue it could severely limit further attempts to obtain clarity on measures of dendritic structure. Future studies either need to employ mixed effects models when analyzing dendritic measurements, or average the measurements from a single animal and insert those averages into a simple linear model, which leads to a quite small upward shift of *p*-values compared to using a mixed effects model [60].

A way to limit the impact of this problem would be to use absolute changes in dendritic measurements rather than SMD. However, massive inter-study variations in dendritic measurements preclude this approach. In fact, the reported spine densities exhibited a variation of two orders of magnitude presumably due to errors in labeling of the graphs. Even when correcting obviously erroneous labels, differences in spine density varied over threefold consistently in several different brain areas, even when utilizing the same staining and imaging techniques. This suggests that not all these findings can be considered reliable, or else that there exists massive biological variation in dendritic architecture that would be hard to reconcile with the current theories of brain development and function. Additionally, the methods employed for quantifying the dendritic tree are often described in a cursory manner, making it challenging to ascertain the underlying causes of these discrepancies. Only a single study reported assessing tissue shrinkage during processing, which could explain part of this variation. Further, many studies did not specify criteria for selecting dendrite branches for spine counting. In particular, not establishing a minimum length of branch for analysis could skew the data if too short stretches were selected. Finally, we also noted that several of the representative images made it clear that spine detection in many cases was not a trivial task, and inter-observer differences when manually counting spines are another plausible source of variation.

Heterogeneity was substantial overall, and in all subgroups large enough to reliably assess it. Some of this is likely methodological, as discussed regarding the spread of dendritic measurements. But there are several plausible sources of biological variety. It is possible that SD has differing effects on dendritic structure in different brain regions and/or in different strains and species of rodents. However, stratifying for species did not reduce heterogeneity, nor did stratifying per brain region reduce it in any subgroup large enough to meaningfully interpret. We cannot conclude whether there exist substantial differences in response to SD across these groups. Stress levels in experimental animals varies by many factors, including details of their housing [61] and even the sex of the experimenter [62]. These factors were seldom described let alone controlled for. Given the above-described effects of stress on dendritic structure, differing levels of stress could explain the variation in response to SD protocols.

Just as stress was rarely measured, the effectiveness of the SD protocols was largely assumed. Without monitoring, it is hard to know what proportion of mice found ways to outwit the automated SD protocols to steal moments of sleep. While an experimenter has eyes on the mice during gentle handling, these experiments last for 5 hours or longer. It was generally not described whether the experimenter was alone in administering gentle handling. In our experience, maintaining attention for 5 hours or longer is not trivial, and lapses in attention could well allow mice stolen moments of sleep. Thus, possible variation in SD efficiency is another plausible cause of heterogeneity.

With the above in mind, the below discussion of outcomes should be taken as hypothesis-generating; we assess that we currently lack the requisite studies to make firm conclusions about this important issue. (And we can draw no conclusions when it comes to females apart from the fact that they are not being studied: virtually all animals in these studies were males. Here we merely note that the need for expanding preclinical neuroscience to encompass females remains great.)

SD of 24 hours or less did not have any significant effect on dendritic spine density or dendritic length. Only three studies reported dendritic length after acute SD; we go no further than to state that we currently do not possess sufficient evidence for any effect on dendritic length from short-term sleep loss. Eleven studies reported spine density after acute SD, justifying a claim that large (> 1 SMD, ~30%) changes in spine density after acute SD are unlikely, but we lack sufficient power to rule out smaller, possibly effects.

Meanwhile, chronic (> 72 hours) SD led to significant decreases both in dendritic length and spine density. Intriguingly, the effect size was similar to SD of 3–6 days, 1–2 weeks, or 3–4 weeks. This suggests either that the detrimental effects of sleep loss plateau rather than accumulate, possibly due to compensatory mechanisms. While chronic SD resulted in significant differences in dendritic measures from control animals, this effect was not statistically significant from that of acute SD. Time will tell whether further studies will, through increased power cause this difference to become significant, or cause the difference to disappear. We will only briefly indulge in speculating on possible explanations for why the chronic SD protocols may have a greater effect than the acute SD protocols.

Interestingly, there is a difference in the effects of acute versus chronic SD on both transcription and translation of plasticity-related genes [63]. Brain-derived neurotrophic factor (BDNF) is a central regulator of plasticity and studies have shown that short-term SD results in upregulation of BDNF as well as having a short-term positive effect against depression. Longer SD instead results in a reduction of BDNF while also increasing stress vulnerability [64]. BDNF can increase plasticity by increasing the release of glutamate and the single channel opening probability of the N-methyl-D-aspartate channel thus upregulating the frequency of excitatory postsynaptic currents (EPSCs) [65, 66]. BDNF also plays a central role in enhancing structural plasticity [67]. Hence, differences in BDNF levels after acute versus chronic SD is a promising molecular pathway for explaining why we found significant losses of dendritic spines after chronic SD but not after acute SD. Higher BDNF levels following acute SD would also likely contribute to higher spine density. Such an effect was not observed in this study, but neither can it be ruled out. SD also increases the expression of pro-inflammatory cytokines such as tumor necrosis factor (TNF) -α and interleukin (IL)-1b [8]. TNF-α is a recognized sleep regulatory molecule that promotes NREM sleep duration [68]. TNF-α expression is at the highest concentration when it is time to fall asleep and increase further by SD [69]. Following chronic SD TNF-α levels remain elevated compared to animals exposed to normal sleep cycles [70]. TNF-α has been shown to potentiate glutamatergic signaling, and to through these mechanisms play a role in synaptic upscaling [71]. However, sustained TNF-α upregulation can result in activation of microglia [72] and excessive synaptic pruning, resulting in synaptic loss [73].

Another possibility is that the difference between the acute SD experiments and chronic SD experiments is related to methodology rather than duration. Acute SD was predominantly performed by gentle handling, while chronic SD was predominantly achieved through variations on the platforms in water approach. We thus cannot conclude from this study whether losses of dendritic spines are specific to prolonged duration (> 3 days) of SD, or specific to the platforms in the water method. Gentle handling, while not stress-free, has been shown to be less stressful than both rotating rod and platforms in water approaches to SD, however, the level of stress induced by gentle handling is highly dependent on the specifics of the person performing it, and the preceding handling protocol [62, 74]. Habituating rodents to the gentle handling protocol by brief daily handling prior to the experiment is a common practice, and has been shown to result in lower stress levels [75]. In fact some studies suggest that rodents do not habituate to handling, and that repeated handling can lead to increased stress [76, 77]. Crucially for this study, handling can also cause sleep loss in the period leading up to the experiment [76]. As the handling protocols were uniformly poorly described, this is another source of significant and difficult to account for heterogeneity between the studies in this review.

Apart from blocking REM sleep the platform method also results in prolonged time periods of social isolation, a risk of fear, and stress as well as hypothermia. None of the studies quantified the number of times mice fell into the water or other similar characteristics. Thus, the possibility exists that the possible difference in the effect of acute or chronic SD are to a large part driven by varying amounts of stress accompanying the respective methods. Chronic stress is known to increase the expression of cytokines in the brain [78], which can increase synaptic pruning [79] as well as affect long-term potentiation and long-term depression[80]. As very few studies measured stress levels, it is hard to know if it had an effect. Additionally, gentle handling aims for total SD while the platform method is intended to specifically disturb REM sleep. The atonia accompanying REM sleep is what is intended to causes the mouse to fall into the water; NREM sleep can after an adjustment period, occur in close to, if not entirely, normal amounts [81]. This could explain the above findings if REM sleep promoted the formation or maintenance of spines while NREM sleep instead promoted pruning. There is to our knowledge few studies supporting such division of labor, if anything REM sleep has been implicated in spine elimination [82], and other explanations should be pursued before considering this speculative venue.

A significant effect on spine density was found only in CA1. This could be explained by the CA1 being the most studied area, and thus the only brain area enjoying a modicum of statistical power. That the hippocampus has received a lot of focus is not unjustified as it is a region considered to be highly sensitive to SD [63]. However, when we look at the individual studies looking at non-hippocampal regions it is clear that the absolute majority of those studies had CIs that included no effect. Further studies would be needed before concluding whether this reflects a biological difference or insufficient power regarding other brain areas.

This study does not offer support for SHY, but due to issues of underpowered studies, improper statistical handling, and insufficiently documented methodology neither does it oppose SHY. We can conclude that chronic SD by platforms in water reduces both CA1 dendritic length and spine density, but we cannot conclude what mechanism drives this effect. We can rule out neither biologically relevant decreases nor biologically relevant increases in dendritic length and spine density after acute SD. We can justifiably argue that any increase in spine density after acute SD is unlikely to exceed 1 SMD (~20%).

The issues that prevent the current body of work from arriving at a firm conclusion are not insurmountable. Concretely, we identify the following as the most crucial actionable items:

- Proper statistical handling of dendritic structure data, either averaging per mouse or using mixed effects models.
- Larger sample sizes, guided by power analyses or by the power simulation in this paper, and methodology chosen to conform to other studies to allow future pooling.
- Stricter and more well-documented image analysis.
- More studies specifically on acute SD.

The methodological checklist developed for this study can serve as a useful tool when designing future studies. By following these recommendations, we are confident we can soon revisit this topic with more conclusive findings.

## Supplementary Material

zsaf146_suppl_Supplementary_Material

## Data Availability

All data used to generate this manuscript is available from the authors.
