# Supplementary material for: Sleep deprivation and dendritic architecture: a systematic review and meta-analysis
Source: Sleep. 2025 Jun 4;48(9):zsaf146. doi: 10.1093/sleep/zsaf146 (PMC12417018; doi:10.1093/sleep/zsaf146)
Supplement: zsaf146_suppl_Supplementary_Material [file zsaf146_suppl_supplementary_material.docx]

# Sleep deprivation and dendritic architecture: a systematic review and meta-analysis

Supplementary information

Alvin TS Brodin^1^, Franziska Liesecke^2^, Julia Spielbauer^1^, Tobias E Karlsson^1^

^1^ Department of Neuroscience, Karolinska Institutet, Sweden

^2^ Department of Plant Biology, Swedish University of Agricultural Sciences, Sweden

Correspondence

[alvin.brodin@regionstockholm.se](mailto:alvin.brodin@regionstockholm.se)

[Tobias.karlsson@ki.se](mailto:Tobias.karlsson@ki.se)


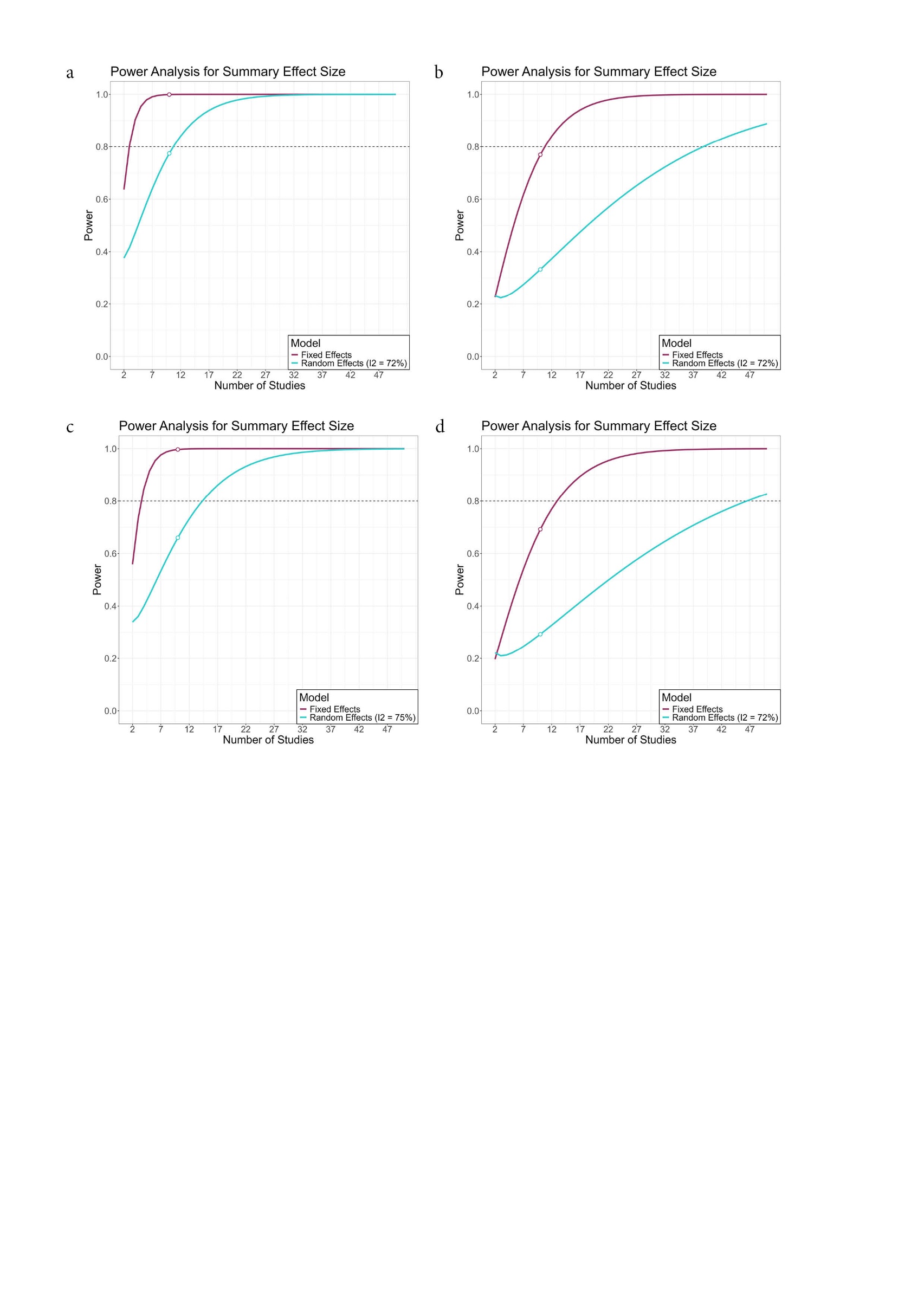


**Figure S1. Power curves.** A. Power curve for SMD=1, dendritic length. B. Power curve for SMD=0.5, dendritic length. C. Power curve for SMD=1, spine density. D. Power curve for SMD=0.5, spine density. Purple line indicates fixed effects model, teal line indicates random effects model.


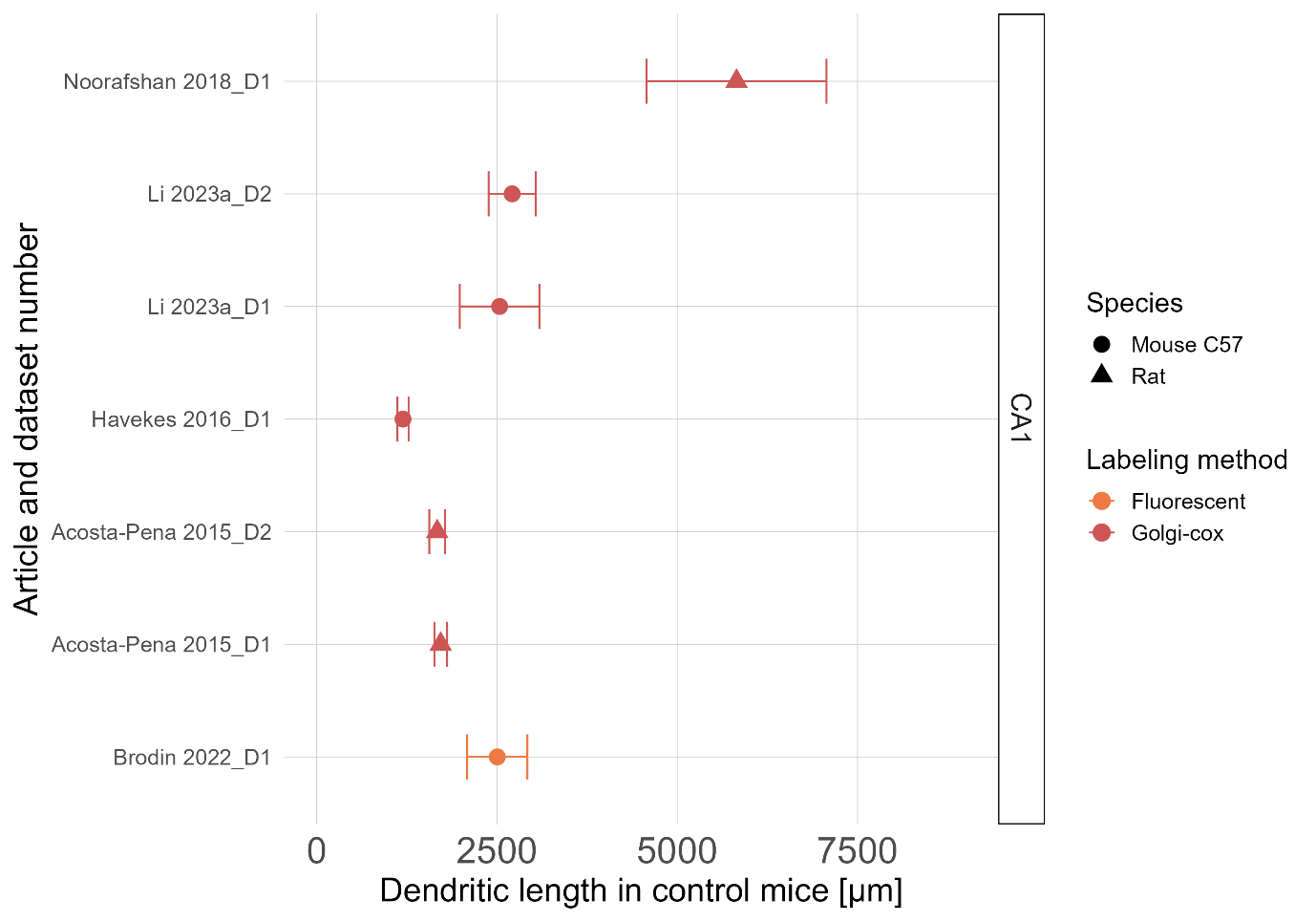


**Fig S2. CA1 dendritic length in control mice.** Average dendritic lengths and 95% confidence intervals across control animals in included CA1 studies. Species are marked by the symbol. Labeling method is reflected by color.


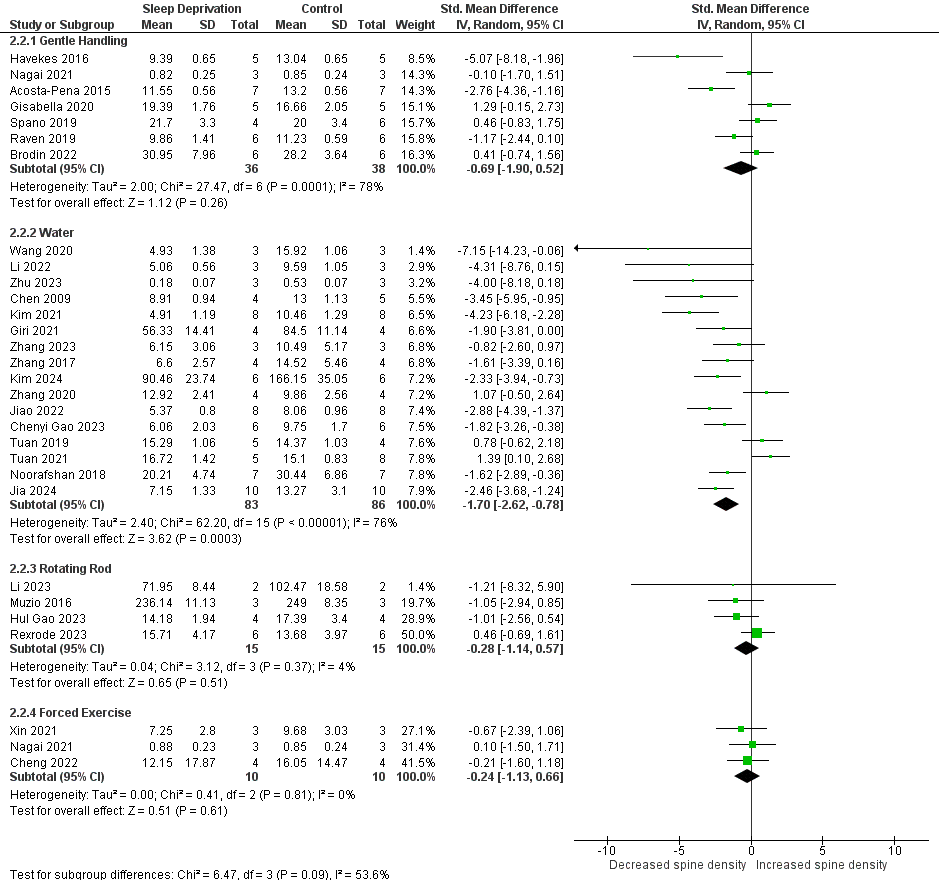


**Figure S3. Forest plot of spine density by sleep deprivation method.** Studies ordered within each subgroup with the highest weighted studies towards the bottom. The size of the green dot reflects study weight, error bars indicate 95% confidence interval, black diamonds indicate 95% confidence interval of summary effect.


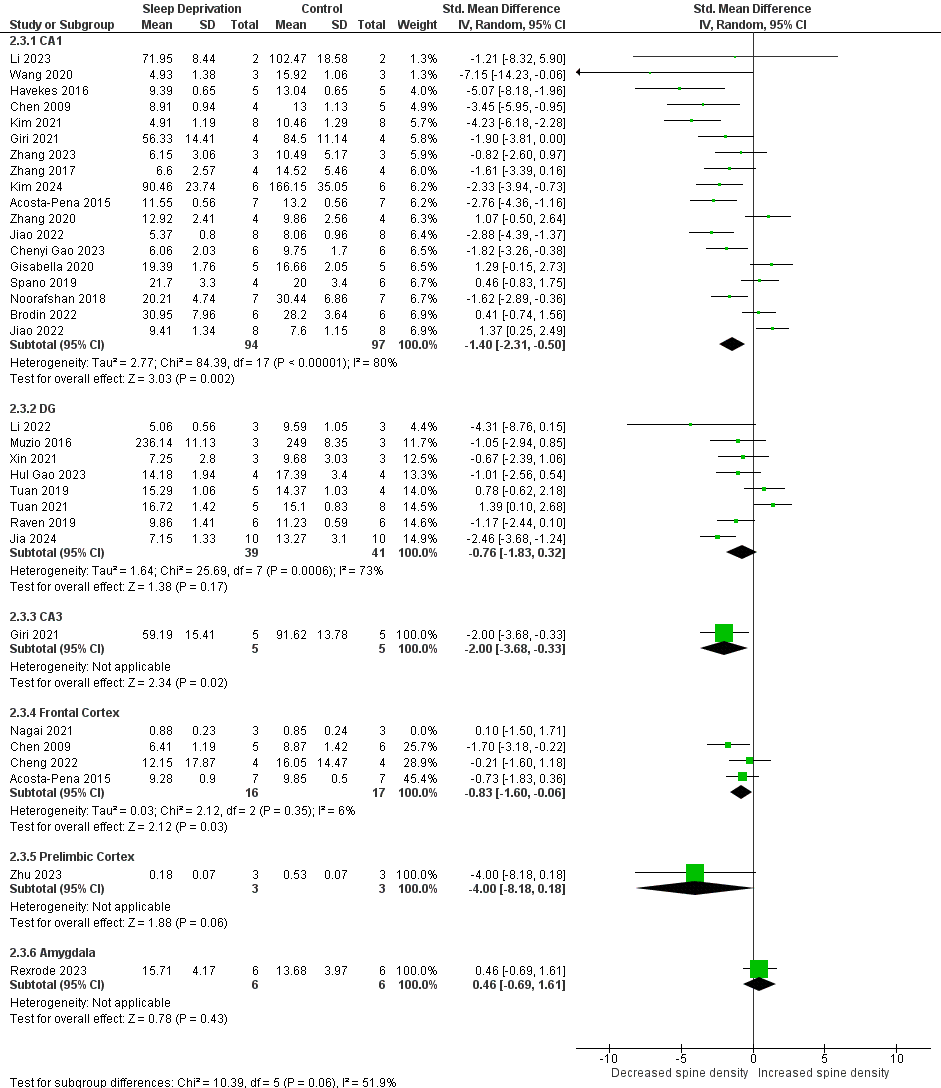


**Figure S4. Forest plot of spine density by brain region.** Studies ordered within each subgroup with the highest weighted studies towards the bottom. The size of the green dot reflects study weight, error bars indicate 95% confidence interval, black diamonds indicate 95% confidence interval of summary effect.


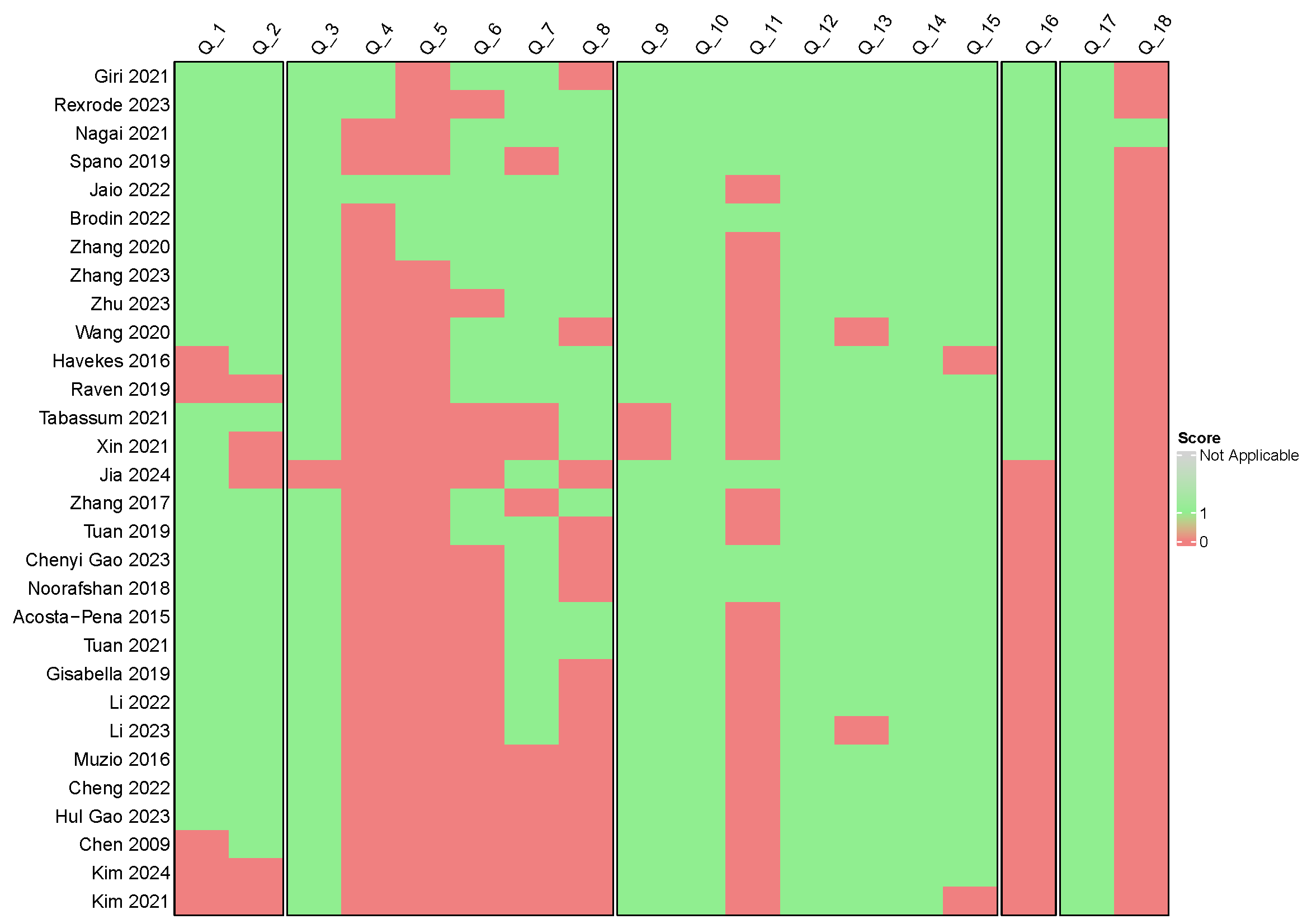


**Figure S5. ARRIVE checklist.** ARRIVE reporting checklist. Green boxes indicate positive, and red boxes indicate a negative answer to the question, with gray boxes indicating the question is not applicable. Detailed questions (Q1-Q18) can be found in the ARRIVE checklist (30).


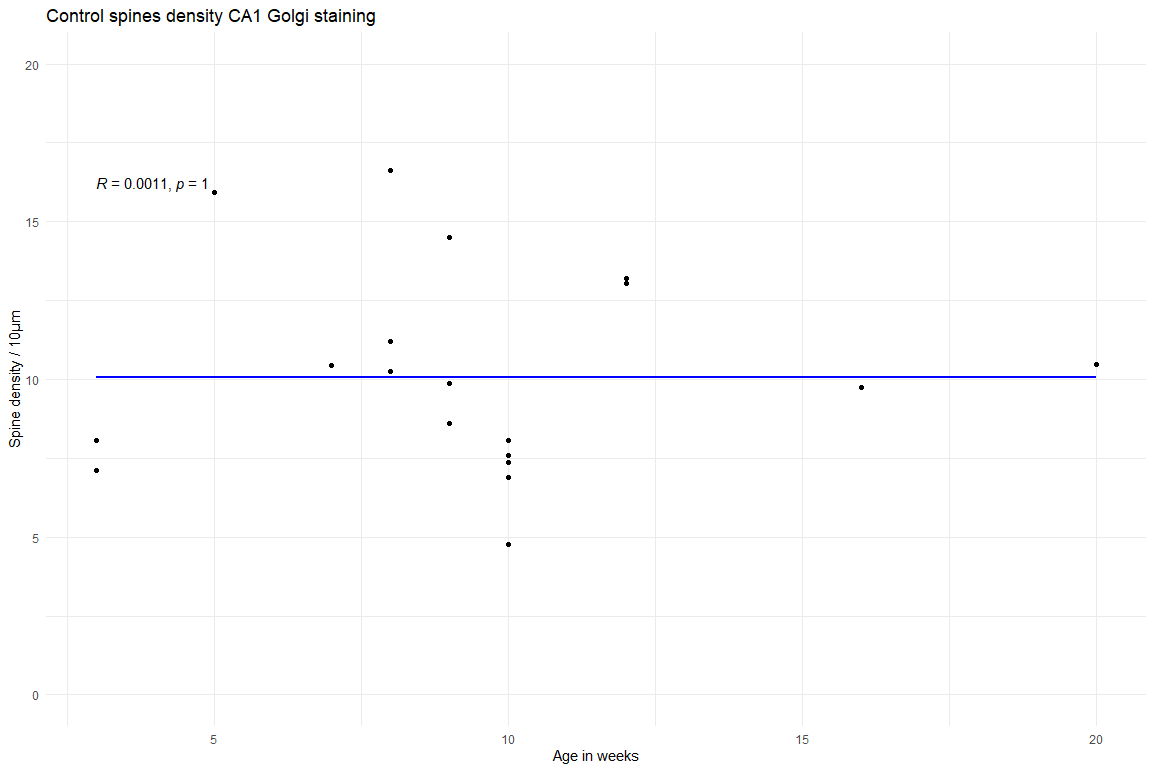


**Figure S6. Correlation between age and spine density**

Spine density plotted vs Age in weeks. One dataset with age 80 and one dataset with spine density over 20 was removed. If included they gave a small but significant increase in spine density with age.

**Figure S7. Detailed methodological checklist.** Heatmap showing the results of the checklist analysis. Gray means not applicable, green equals Yes and red equals No, light green is yes after communication with the authors of the papers. Thicker line separate the different clusters. Studies mark with bold are acute and underlined text marks datasets that had both an acute and a chronic group. Dashed blue line shows boundary between studies that used either acute, chronic or both types of sleep deprivation.


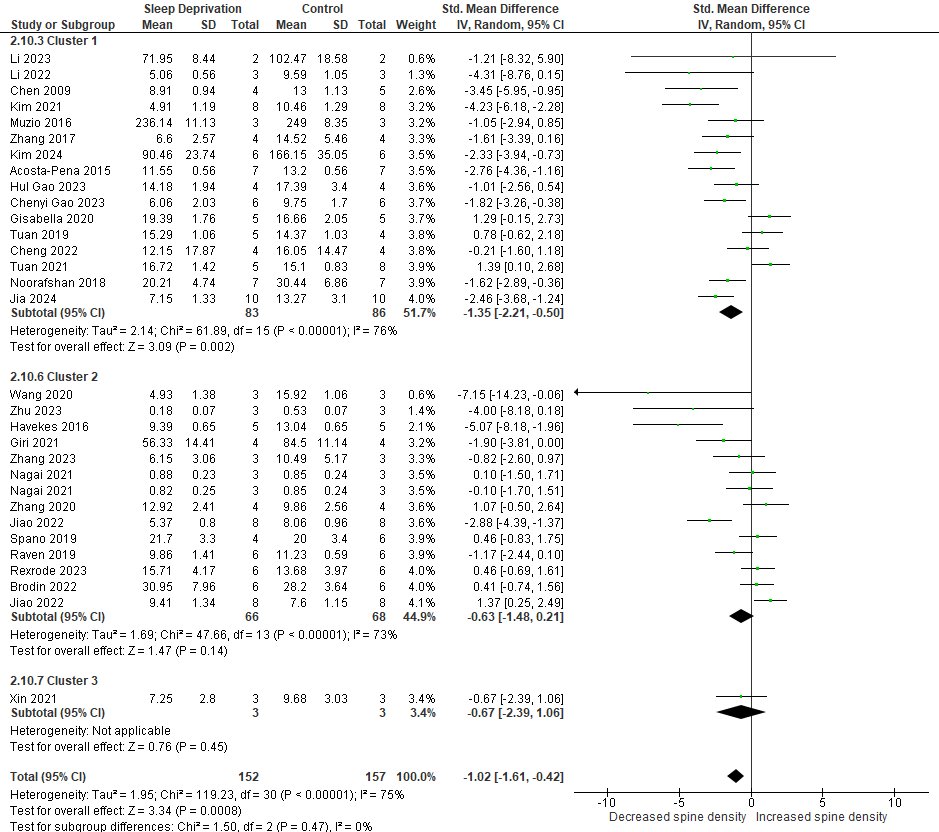


**Figure S8. All included studies sub-grouped by cluster with the highest weighted studies towards the bottom.** The size of the green dot reflects study weight, error bars indicate 95% confidence interval, black diamonds indicate 95% confidence interval of summary effect.

**
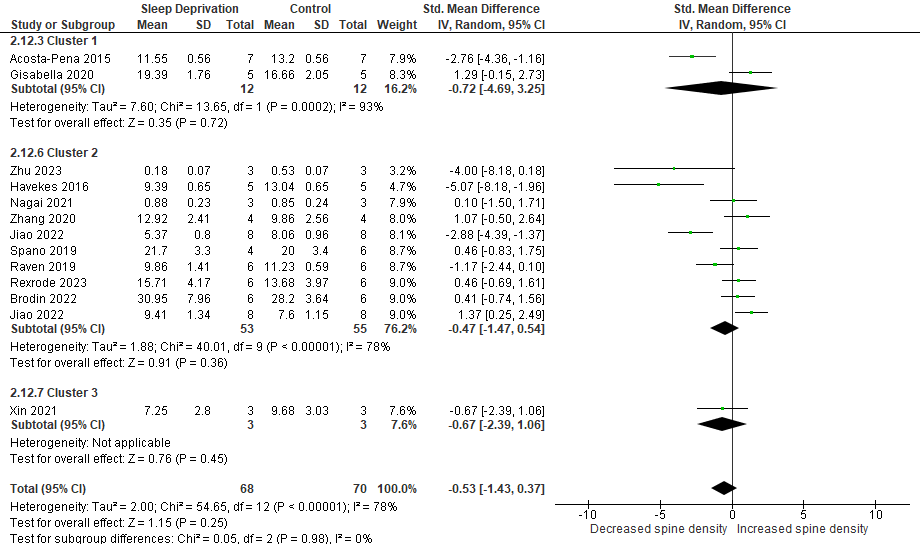
**

**Figure S9. Acute studies sub-grouped by cluster with the highest weighted studies towards the bottom.** The Size of the green dot reflects study weight, error bars indicate 95% confidence interval, black diamonds indicate 95% confidence interval of summary effect.

**
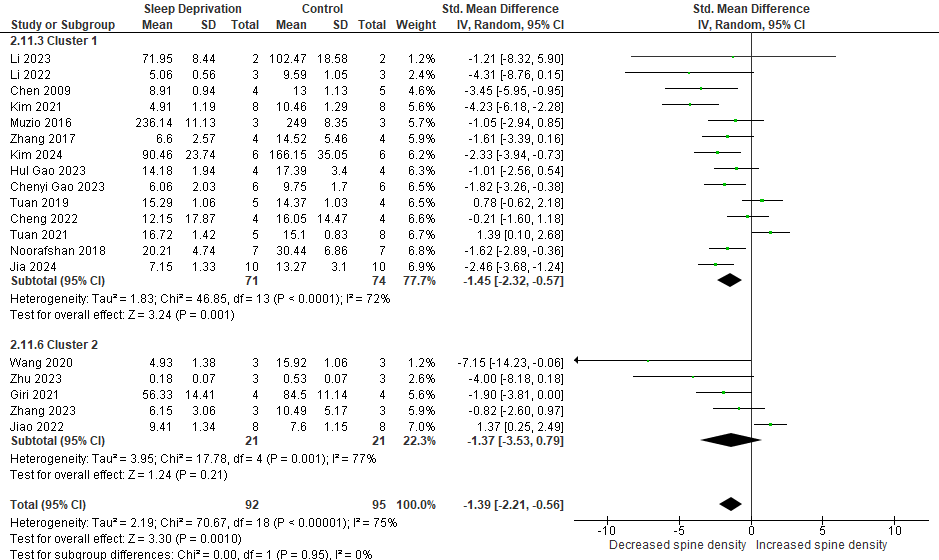
**

**Figure S10. Chronic studies sub-grouped by cluster with the highest weighted studies towards the bottom.** The size of the green dot reflects study weight, error bars indicate 95% confidence interval, black diamonds indicate 95% confidence interval of summary effect


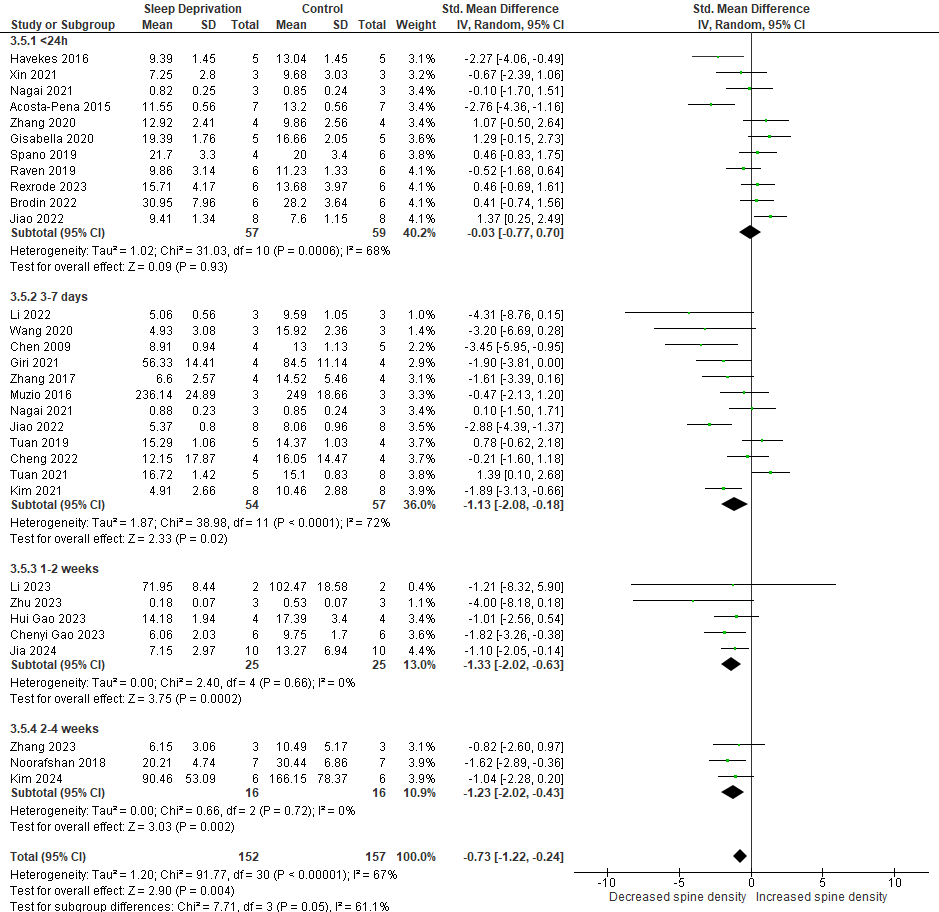


**Figure S11. Forest plot of spine density by duration, assuming neurons treated as dependent.** Studies ordered within each subgroup with the highest weighted studies towards the bottom. The size of green dot reflects study weight, error bars indicate 95% confidence interval, black diamonds indicate 95% confidence interval of summary effect.
